# Cautionary response strategy and impairment of post-conflict response selection underlie age-related differences in a location-based Stroop task

**DOI:** 10.3389/fpsyg.2025.1565846

**Published:** 2025-07-21

**Authors:** Ali Pournaghdali, Teal S. Eich

**Affiliations:** Leonard Davis School of Gerontology, University of Southern California, Los Angeles, CA, United States

**Keywords:** attention, perception, aging, inhibition, decision

## Abstract

**Introduction:**

Research suggests that older adults have deficits in selective attention, a cognitive process often queried through the Stroop task. To tease apart whether this is due to failures to inhibit distracting information or to upregulate attention towards target information, younger and older adults completed a task called the Shape Stroop.

**Methods:**

In this task, participants had to name the color of a shape that was occluded by another shape. Critically, congruent or incongruent Stroop words were placed in either the target shape, the occluding (distractor) shape or in the background. We first modeled performance as a function of age-group, Stroop word congruency, and location.

**Results:**

The results indicate that older adults were more accurate but slower than younger adults to choose the correct shape color. For both younger and older adults, incongruent words induced slower reaction times when words were in the target location, indicating age-invariance in the Stroop effect. To further probe how early and/or late attentional processes contribute to performance and to interrogate the decision strategies adopted across different conditions, we also fit the dual-stage two-phase model of selective attention to our data.

**Discussion:**

Our results indicate that older adults tend to be more cautious and require more information before choosing a stimulus to attend to or making a decision. Although older adults’ ability to inhibit irrelevant information seems intact, they show signs of slower information processing in the later stages of attentional processing.

## Introduction

Selective attention is a critical element of day-to-day cognitive functioning, and is composed of two functionally separate but interrelated classes of processes (Posner, 1980). On the one hand are facilitatory processes that amplify the task-relevant object; on the other are processes that inhibit responses to task-irrelevant objects or distractors. One experimental task frequently used to query these processes is the Stroop (Stroop, 1935), wherein participants are presented with color-words and must name color of the ink. Ubiquitously, participants show slowed reaction times and increased error rates when they have to name colors that are incongruent (“blue” printed in red ink) relative to congruent (“blue” printed in blue ink) to the color-word. Considerable research indicates that the Stroop effect is larger in older relative to younger adults (Bugg et al., 2007; Houx et al., 1993; Ludwig et al., 2010). The locus of this age-related effect, however, is debatable: a large meta-analysis showed that age-related differences were attributable to general age-related slowing (Verhaeghen and De Meersman, 1998). However, other research has found that differences in Stroop interference effects are attributable to age-related deficits in the ability to inhibit the habitual response (in this case, reading the word), even after accounting for age-related declines in speed of processing (Davidson et al., 2003; Hasher and Zacks, 1988), a deficit thought to reflect general age-related declines in response inhibition (Eich et al., 2017, 2018; Hasher et al., 2007; Hasher and Zacks, 1988).

In the traditional Stroop task, the target and distracting information are spatially overlapped, and are usually presented in the center of the visual field. However, in the real world, the effects of facilitatory or inhibitory aspects of selective attention may depend on where target and distracting information occur. When driving down the street, for example, stimuli that must be attended to (the color of the traffic light) or inhibited (billboards) may be close, while others may be further away; some may be relevant (the pedestrian in the crosswalk), whereas others may be irrelevant (the car pulling out on the other side of the street) to the task at hand. To function optimally, one must be able to guide attention toward task-relevant stimuli and turn attention away from and ignore the task-irrelevant ones, regardless of where these occur. However, there may be age-related differences in the ability to facilitate relevant information and inhibit irrelevant information that is affected by where this information occurs (e.g., Castel et al., 2003; Geerligs et al., 2012; McLaughlin et al., 2010; also see Beurskens and Bock, 2012). For example, Geerligs et al. (2014) showed that older adults are less efficient at inhibiting irrelevant information when the target and irrelevant information are far apart. In this study, older and younger participants were cued about the location of a target letter (e.g., “A”). During the testing, the target was presented in the cued location (relevant location) or in the location other than the cued location (irrelevant location). In the control condition, no target was presented. Whereas reaction times were comparable between the two groups when the target was presented in the relevant location, older adults were slower at selecting the target in the irrelevant location than were younger adults. These results indicate that older adults may be more prone to interference from non-foveal information and have difficulties inhibiting irrelevant information when there is a spatial separation between the target and the irrelevant information. Hence, spatial context may modulate age-related differences in inhibitory and facilitatory processes involved in selective attention.

In order to understand the role of spatial context of these two aspects of selective attention, Wühr (2007), Wühr and Frings (2008, 2009), and Wühr and Waszak (2003) developed a spatial attention task that capitalized on findings from the traditional Stroop task. In their task (which we will refer to as the Shape Stroop task henceforth), participants named the color of a shape that was occluded by a different shape of a different color. Color-words (always printed in black), which were either congruent or incongruent to the target shape’s color, were placed in the to-be-attended shape (target condition), the to-be-ignored shape (distractor condition), or the background (background condition). Through this design, both attentional facilitation and inhibition of items in differing spatial locations can be examined: to the extent that attentional resources are upregulated toward the target, congruent words placed in the target should facilitate target color identification, whereas incongruent words should hinder it. On the other hand, to the extent that information presented outside of the target shape is inhibited, Stroop effects from words placed anywhere other than the target should be mitigated. Thus, intact attentional focus predicts strong target-based Stroop effects (facilitation when the color word and target shape are color-congruent and interference when they are incongruent), whereas intact inhibitory processing predicts smaller non-target Stroop effects. Indeed, this is exactly what Wühr and Waszak (2003) found, suggesting that younger adults can successfully upregulate attention toward the relevant shape, and simultaneously inhibit information in non-relevant, spatially distinct locations.

Here, we will leverage this task to explore potential age-related differences in both facilitatory and inhibitory processes involved in selective attention. We first examined overall performance based on accuracy rates and reaction times to incongruent vs. congruent trials as a function of age-group and location using the traditional inferential approaches. Based on the existing literature, we expected to observe the largest age-related difference in the Stroop effect when color-words were presented in the target shape. However, we also expected to observe age-related differences in the Stroop effect when the color-words were presented in the distractor shape or in the background, but with a lower magnitude than when the color-words were presented in the target shape.

In addition, we will use a formal computational model to investigate the underlying age-related differences in how decisions that require evidence accumulation are made over time within each trial, and the thresholds that produce a choice. The drift diffusion model (DDM; Busemeyer and Townsend, 1993; Ratcliff, 1978; Ratcliff and McKoon, 2008) has been used to compare age-related differences in reaction time data from different tasks, including old/new item recognition, old/new paired associates recognition, letter discrimination, brightness discrimination, numerosity discrimination, and lexical decision (e.g., McKoon and Ratcliff, 2012, 2013; Ratcliff et al., 2001, 2006; Ratcliff et al., 2004b; Ratcliff et al., 2004a; Ratcliff and McKoon, 2015). DDM allows for an understanding of how much of the delay is due to processing conflict, how much is due to general caution, and how much is just about perception and motor execution. DDM, however, does not account for the presence of task-relevant and task-irrelevant information. At least three extensions of DDM have been proposed that take into account both task-relevant and task-irrelevant information: the diffusion model for conflict tasks (DMC; Ulrich et al., 2015), the shrinking spotlight model (SSP; White et al., 2011), and the dual-stage two-phase model of visual attention (DSTP; Hübner et al., 2010; Hübner and Pelzer, 2020). The last two models, the SSP and the DSTP, are specifically built based on theories of attention to explain the pattern of reaction time data in the flanker task (Eriksen and Eriksen, 1974).

Both the SSP and the DSTP models assume that a cognitive/perceptual decision is the final product of a process that starts from the onset of the stimulus and accumulates evidence in favor of one of two possible decisions. Accordingly, both models postulate that the level of interference from irrelevant information does not remain constant throughout a trial. They do so by proposing different mechanisms that underlie response selection in tasks such as the Flanker and Stroop. Whereas the SSP assumes one evidence accumulation process in which there is a gradual reduction of the interference effect during a trial, the DSTP suggests the presence of two functionally different but interacting evidence accumulation phases, in which task-irrelevant stimuli only impact the first phase. Despite the difference between the SSP and the DSTP, research suggests that the two models perform similarly when fit to the data from the Flanker task (Evans and Servant, 2020, also see White et al., 2018) and can account for the presence of interfering information in the decision-making processing (Evans and Servant, 2020; Hübner and Pelzer, 2020). Hence, the decision about which model to use depends on the question at hand. In our case, differences in performance across locations in the Shape Stroop task may be due to dissociable processes, one facilitatory and the other inhibitory, which are involved in early vs. late stages of selective attention (see Parris et al., 2022; also see Gratton et al., 1992) and may be differentially affected by age (e.g., see Spieler et al., 1996). Accordingly, as we were interested in potential age-related differences in facilitatory and inhibitory processes involved in different stages of selective attention as well as potential age-related differences in the response strategies used to make a cognitive decision in the Shape Stroop task, we used DSTP (Hübner et al., 2010; Hübner and Pelzer, 2020). current study, we chose to use the DSTP rather than the SSP model.

According to the DSTP, an observer’s response selection happens in two phases. The first phase is constituted of a noisy evidence accumulation process that is regulated by the results of an early attentional filter: the sensory signal that passes through this early attentional filter determines the rate of evidence accumulation in the first phase of response selection. Because this early filter is not optimal, both task-related (the target’s color) and task-irrelevant information (the color-word), influence the evidence accumulation process in the first phase of response selection. Hence, we quantify this evidence accumulation in the first phase using two parameters, namely$\;\mu_{\text{Target Color}}$ and $\mu_{\text{color}-\text{word}}$, which represent the level of influence of target color and color-word on the rate of evidence accumulation, respectively.

At the same time as the onset of the first phase of response selection, a competing late stimulus [attentional] selection stage begins, which is constituted of a different noisy evidence accumulation process responsible for selecting the color of the target-shape solely based on its identity. The rate of late evidence accumulation is represented by the parameter $\;\mu_{\mathrm{SS}}$.

**Figure 1:**
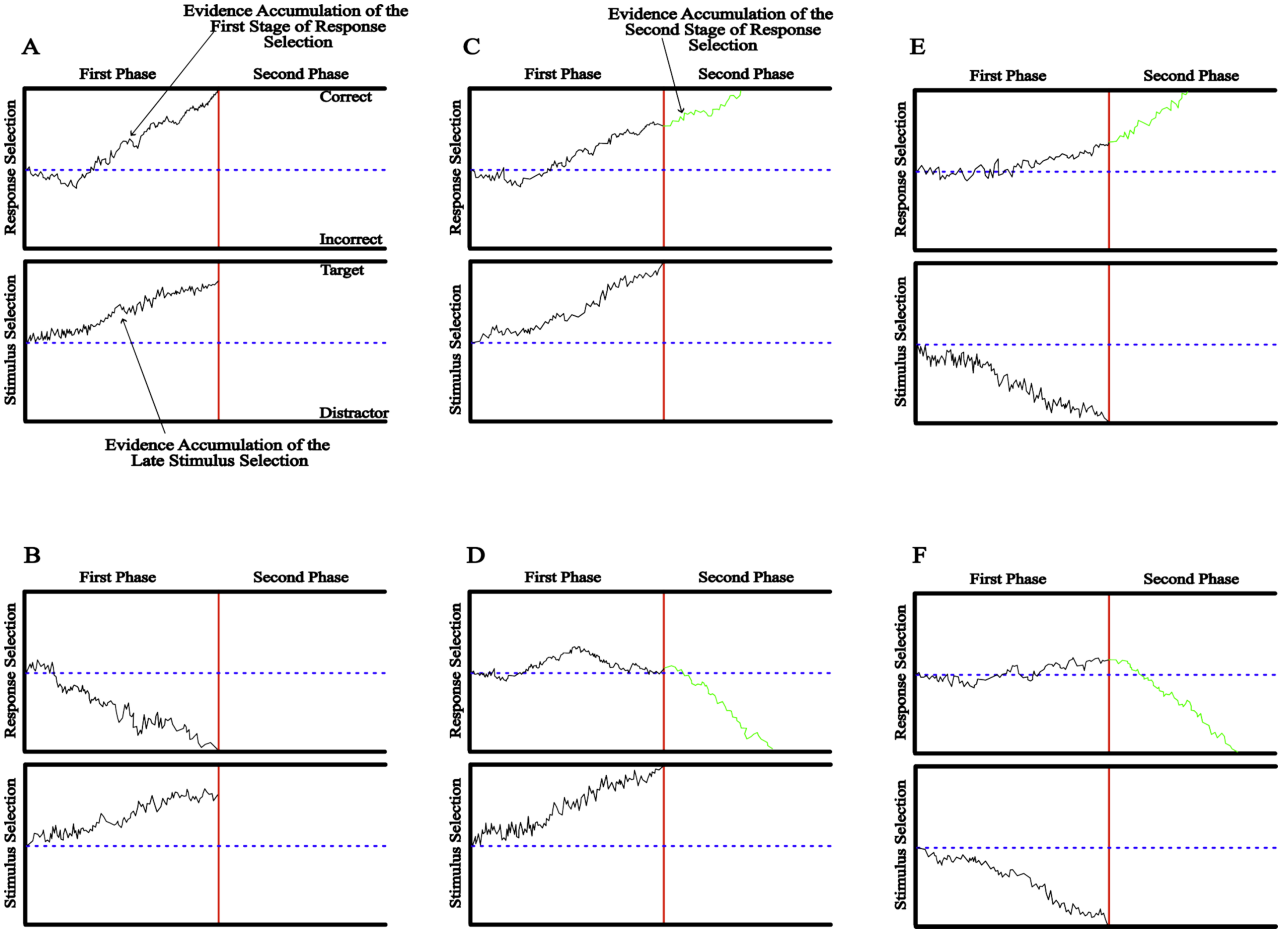
The DSTP model of selective attention. In each graph, the top panel represents two phases of response selection, and the lower panel represents the late stages of stimulus selection. The x-axis of each panel represents time, with zero representing stimulus onset. The four solid black horizontal lines within each graph represent a response or a stimulus selection boundary. The red vertical line represents the time point on the x-axis, where the accumulation of evidence in the first phase of response selection (top panel of each graph) or in the late stage of response selection (top panel of each graph) reaches a boundary. Note that the first phase of response selection and the late stimulus selection processes compete to reach a boundary. Depending on the outcome of this competition, six different patterns can occur. If the evidence accumulation responsible for the first phase of response selection reaches the boundary first, the participant initiates a response that can be correct **(A)** or incorrect **(B)**. On the other hand, if the evidence accumulation responsible for the late stimulus selection reaches the boundary first, the first phase of response selection is terminated, and the second phase of response selection begins, which is based on the input of the late stimulus selection stage. In this case, four possible outcomes can occur. The late stimulus selection processing may choose the target **(C,D)** or the distractor **(E,F)**. Although the second phase of stimulus selection most likely chooses the same stimulus as the late stimulus selection processing **(C,F)**, it may instead choose the other stimulus **(E,D)**.

Evidence accumulation processes in both the first phase of response selection and the late stage of stimulus selection compete against each other to reach a boundary. If the evidence accumulation responsible for the first phase of response selection reaches the boundary first (Figures 1A,B), then the observer initiates a response, which is strongly influenced by the color-word. If the color-word is incongruent to the color of the target-shape, an error is more likely to occur (Figure 1B). However, if the evidence accumulation responsible for the late stimulus selection reaches the boundary first (Figures 1C–F), the first phase of response selection is terminated, and a new, second phase of response selection begins, which is comprised of a noisy evidence accumulation process that is based on the input of late stimulus selection stage.

In combination, the traditional frequentist analyses plus the DSTP mathematical model will allow us to quantify whether there are overall performance differences across older and younger adults on the task, different aspects of selective attention processes including age-related effects of attentional facilitation and inhibition, the sources of these differences including in early and/or late attention processes, and potential as changes in response strategies adopted across different conditions.

## Methods

### Participants

Seventy-two older and 75 younger right-handed English-speaking adults with at least a fourth-grade reading level completed 10 computer-based inhibitory tasks, including the Shape Stroop, as a part of a larger study called the Study Of the Factor Structure of Inhibition in Aging (SOFIA). The required sample size for the SOFIA study was determined based on a power analysis for a structural equation model that considered all 10 tasks administered. SOFIA participants were recruited from two ongoing studies at Columbia University (the Cognitive Reserve and the Reference Ability Neural Networks studies; see Eich et al., 2018, 2020; Habeck et al., 2016, 2018, 2019; Stern, 2009), and had previously participated in computer-based experimental cognitive testing in the lab as part of these studies. To participate in the SOFIA study, participants had to have their own laptop or desktop computer, be able to download the Millisecond software platform onto their computers to run the SOFIA study tasks, and be able to dedicate uninterrupted time to complete the study.

Participants with hearing or visual impairment, objective cognitive or functional impairment, diagnosis of a neurologic or psychiatric disorder, or serious memory complaint at the time of recruitment were excluded (see Stern et al., 2014). The Mattis Dementia Rating Scale was used to screen all older participants for dementia, and only participants who scored 135 or higher on this scale participated. All participants were compensated. The Institutional Review Boards of the College of Physicians and Surgeons of Columbia University and the University of Southern California approved this study, and a waiver of informed consent was obtained from all participants. Demographic information for the sample is shown in Table 1.

**Table 1:** Participants’ demographic information.

Younger and older adults		Younger participants	Older participants
Age	Mean (SD)	30.63 (5.52)	72.18 (4.71)
	Range	20–40	65–83
Sex	Male	27	33
	Female	48	39
Education (years)	Mean (SD)	16.51 (1.83)	16.53 (2.44)
Race	White	35	58
	Black or African American	9	12
	Asian	18	0
	More than 1 race	7	1
	Other races	6	1

### Behavioral task and procedure

Participants completed the experiments in their homes using the Millisecond platform. Participants had to follow a detailed set of instructions to download and then install the Millisecond software on their computers, so that the experimental tasks would run locally, thus avoiding any potential differences in network connection speeds or reliability that could affect stimuli presentation rates or response latencies. Participants were required to use a desktop or laptop computer and were instructed to complete the tasks in a quiet environment free from distractions. Before beginning the Shape Stroop task, participants completed two practice blocks to learn the stimulus–response mappings. First, participants classified the color of a square (red, green, blue, or yellow), presented in the middle of the screen, by pressing the appropriate key on the keyboard for the first letter of each color: “R,” “Y,” “G” or “B.” The color-key practice block consisted of 12 trials, 3 for each color, with each presented for 5,000 ms. Then, participants saw two different superimposed shapes and had to indicate the color of the shape that was either on top or underneath. Each practice trial began with a 500 ms presentation of a fixation cross, followed by a 100 ms blank display. Then, a perpendicularly superimposed oval and rectangle were presented for 5,000 ms, followed by a white background for 500 ms. Participants could respond as soon as stimuli onset and until the end of the white background, again by pressing “R,” “Y,” “G” or “B” on the keyboard.

**Figure 2:**
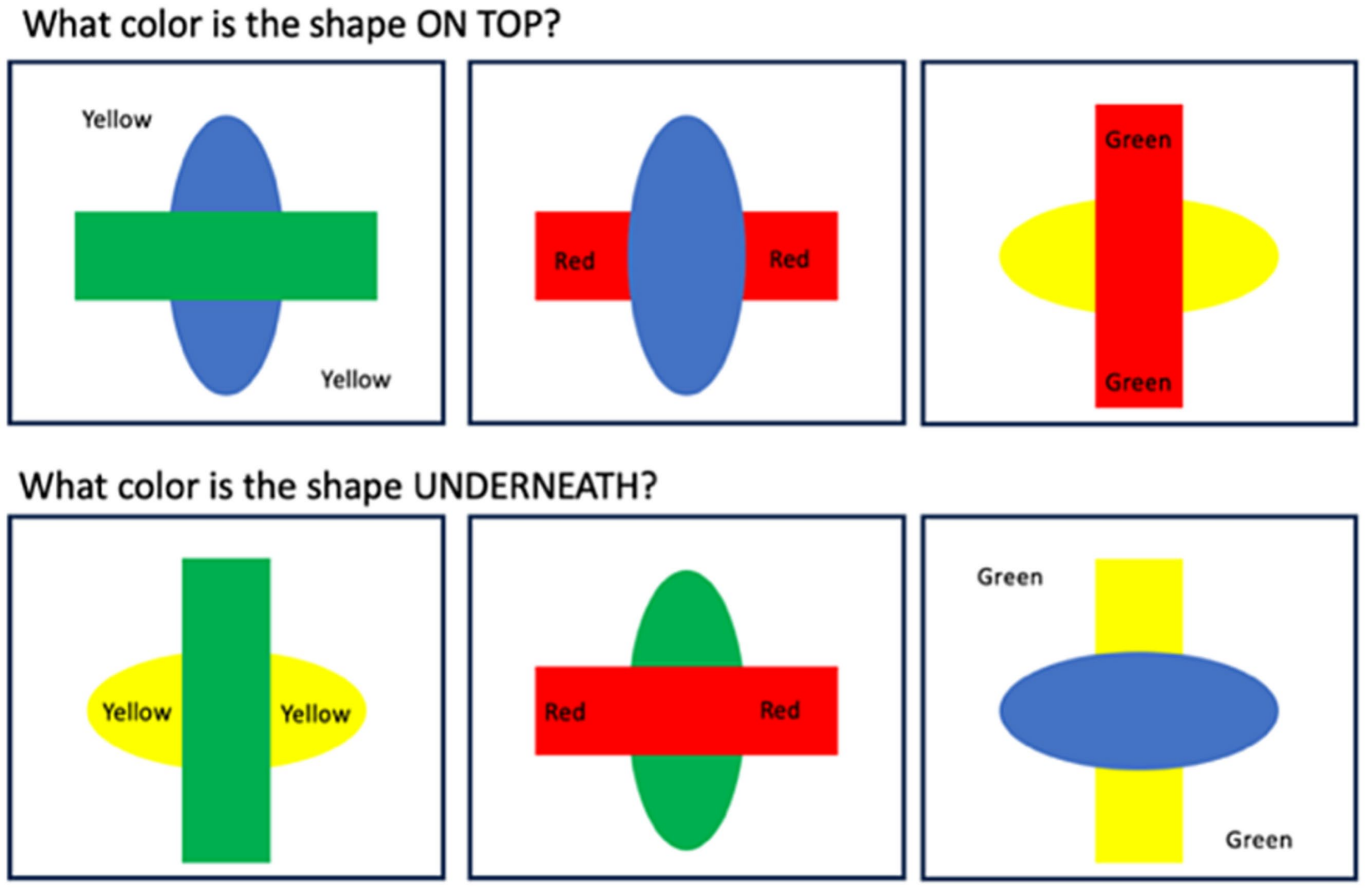
Schematic representation of different conditions in the Shape Stroop Task. Participants had to either indicate the first letter of the color of the shape that was on top, or underneath.

Once participants had completed the training, they then completed 18 experimental blocks, each containing 24 trials, which were identical to the second practice block, except that there were additional color-words (red, green, blue, and yellow), which were always printed in black ink, presented in either the target shape, the distractor shape, or in the background (Figure 2). Participants were told to ignore these words. There were six trials of each color, and the target shape (rectangle or oval) was balanced, as was the target orientation (horizontal or vertical). Within each block, 18 trials contained color-words that were incongruent to the target shape color, and six trials contained color-words that were congruent to the target shape color. The order of blocks and the order of trials within each block were randomized for each participant.

### Analysis

All statistical analyses were performed in R (version 4.0.5), extended with the packages *lme4* (Bates et al., 2015) and *flankr* (Grange, 2016). Behavioral data, estimated model parameters, R syntax used to perform the analyses, and the supplementary materials file are available at: https://osf.io/5tyqb/.

### Stimulus–response mappings

Arbitrary stimulus–response mappings are frequently used to control the position of participant’s fingers on the keyboard in experimental tasks. For example, in computerized Stroop tasks, participants might be instructed to press the F, G, H or J keys using their left middle, left pointer, right pointer, and right middle fingers when they see words printed in red, yellow, green or blue, respectively. However, even with substantial training, age-related differences are often reported for this type of arbitrary stimulus–response mapping (Eich et al., 2018; Vu and Proctor, 2008). Thus, in the current study, we used a less arbitrary stimulus–response mapping, having participants press the R key for Red, the Y key for Yellow, etc. We did not instruct participants on finger position, and thus, it is possible that one key press was more difficult or took longer than another or that this mapping would be easier for one of the age-groups. To ensure that this was not the case, we fit a linear regression to the participants’ reaction times from the first practice block (where participants simply had to classify the color of a square using this stimulus–response mapping) with age-group (young vs. old). Any differences in reaction time between stimulus–response mappings and color, or an interaction with age-group, should be evident here if the stimulus–response mapping was affecting performance inequitably. However, we found no such evidence: Neither the effect of stimulus color nor the interaction between age and stimulus color were significant (*p*s > 0.05).

### Overall performance

Accuracy was analyzed using a Greenhouse–Geisser corrected analysis of variance (ANOVA). In this analysis, we used age-group (young vs. old) as the between-subject factor and congruency (congruent vs. incongruent) and color-word location (background vs. target vs. distractor) as the within-subject factors.

We also analyzed participants’ reaction times for correct trials using a Greenhouse–Geisser corrected ANOVA, with age-group as a between-subject factor and congruency and color-word location as the within-subject factors. We corrected the *p*-values for multiple comparisons in all analyses using Tukey.

Following one of our reviewer’s recommendation, we also fit a generalized linear mixed-effects model (GLMM) to the reaction time data for correct trials, with age group, congruency and color-word location as the fixed factors, random intercepts for each participant, and random slopes for congruency and color-word location. In this model, we applied a gamma distribution with a log link function. The result of this analysis is available at the supplementary materials. Because this analysis yielded a similar pattern of results as the ANOVA-based analysis of reaction time, we will not discuss it further.

We also investigate potential sex differences in reaction time. To this end, we performed a similar Greenhouse–Geisser corrected ANOVA analysis with reaction time as the dependent variable, age-group and sex as between-subject factors and congruency and color-word location as the within-subject factors. Finally, we compared younger and older adults’ level of education using an independent *t*-test. The results showed no difference in the level of education between the two groups.

### Dual-stage two-phase (DSTP) model of the shape Stroop effect

While ANOVAs of accuracy and reaction times provide critical information about potential group-level differences across factors manipulated in the experiment, they cannot help us to understand *why* these group-levels differences may exist. To *decompose* participant’s behavior into latent cognitive mechanisms that enable the exploration of the cognitive processes involved in the task (e.g., how decision evidence is accumulated and how control is engaged), we fit three different DSTP models for each participant’s behavioral data: one DSTP model for the target location, one for the distractor location and one for the background location. For each model, we estimated seven free parameters, as shown in Table 2. $\mu_{\text{Target Color}}$ and $\mu_{\text{color}-\text{word}}$ are components of drift rate in the first phase of response selection, $\mu_{\mathrm{SS}}$ is the drift rate of the late stimulus selection, $\mu_{\mathrm{RS}2}$ is the drift rate of the second phase of response selection, *criterion A* represents the height of response selection boundary, *criterion C* represents the height of late stimulus selection boundary and $t_{\mathrm{er}}$ is a non-decision time representing duration of stimulus encoding. Following White et al.'s (2018) recommendation, we also estimated the interference ratio, which is an index for the level of interference of the color-word relative to the speed of stimulus selection: $\mu_{\text{color}-\text{word}}/\mu_{\mathrm{SS}}$ and represents the magnitude of interference that an irrelevant stimulus induces.

**Table 2:** Parameters of the DSTP Model.

Parameter	Meaning
$\mu_{\text{target}-\text{color}}$	Drift rate for the color of the target-shape during the first phase of response selection
$\mu_{\text{color}-\text{word}}$	Drift rate for the color-word during the first phase of response selection
$\mu_{\mathrm{SS}}$	Drift rate of the of the late stimulus selection
$\mu_{\mathrm{RS}2}$	Drift rate of the of the second phase of response selection
Criterion A	Height of response selection boundary
Criterion C	Height of stimulus selection boundary
$t_{\mathrm{er}}$	Non-decision time
$\mu_{\text{color}-\text{word}}/\mu_{\mathrm{SS}}$	Interference ratio index

To ensure that the estimated parameters of each fitted model do not reflect local minima, we first fit 50 different individual DSTP models with different sets of parameters’ values to each participant’s data and simulated 5,000 trials per each, keeping only the model containing the parameters that minimized the likelihood ratio chi-square ($G^{2}$) statistic (Grange, 2016). We used those estimated parameters to fit a final model to each participant’s individual data and simulated 100,000 trials for this final model. We then compared each parameter across the two age-groups and three color-word locations using a mixed-effect ANOVAs, one for each estimated parameter plus the interference ratio index. Before performing each ANOVA, we removed participants whose estimated parameters were outliers based on predefined bounds (see supplementary materials). For each parameter, we first estimated the interquartile range (IQR), which is the difference between 75th and 25th percentiles. Next, we multiply the IQR by 1.5 and subtracted from 25th percentile as well as added it to the 75th percentile. The resulting two point-estimates were lower and higher bound used to identify and exclude the outliers. Therefore, any values smaller than the lower bound or larger than the higher bound were flagged as outlier and excluded from further analysis. Although the exclusion of outliers was decided before performing the final ANOVA analyses, we also performed each ANOVA analysis again, but with the inclusion of outliers, the results of which are presented in the supplementary materials.

## Results

### Stimulus–response mappings

A linear regression of reaction times in the practice trials with age and color as the independent variables revealed that neither the effect of stimulus color nor the interaction between age and stimulus color were significant (*p*s > 0.05).

### Overall performance

**Figure 3:**
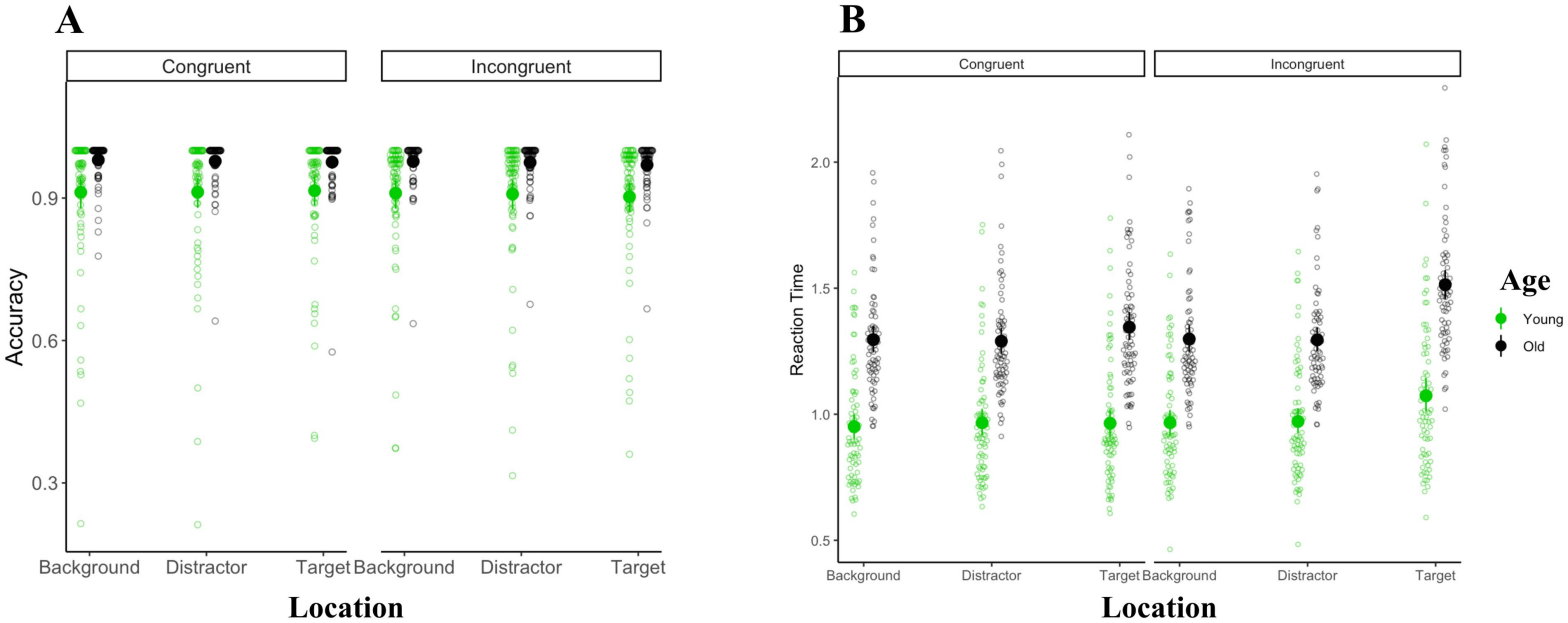
Overall performance results. Panel **(A)** shows the accuracy results and panel **(B)** shows the reaction time results. The mean of reaction time and accuracy within each condition is represented with solid black and green circles for older and younger adults, respectively.

As is shown in Figure 3, the Greenhouse–Geisser corrected ANOVA of accuracy using age-group (young vs. old) as the between-subject factor and congruency (congruent vs. incongruent) and color-word location (background vs. target vs. distractor) as the within-subject factors revealed significant main effects of age [*F*_(1, 145)_ = 15.3, MSE = 0.1, $\eta_{p}^{2}$ = 0.1, *p* < 0.001] and congruency [*F*_(1, 145)_ = 16.1, MSE < 0.01, $\eta_{p}^{2}$ =0.1, *p* < 0.001]. The main effect of word location, as well as any other interaction, were not significant (*ps* > 0.09).

Next, we computed the Greenhouse–Geisser corrected ANOVA of reaction times for correct trials with age-group as a between-subject factor and congruency and color-word location as the within-subject factors. This revealed significant main effects of age [*F*_(1, 145)_ = 89.8, MSE = 0.3, $\eta_{p}^{2}$ = 0.4, *p* < 0.001] such that older adults had slower reaction times overall [older adults: 1.34 s (sd = 0.25); younger adults: 0.98 s (sd = 0.24)], congruency [*F*(1, 145) = 93.9, MSE < 0.01, $\eta_{p}^{2}$ = 0.4, *p* < 0.001] such that reaction times were faster for congruent (mean = 1.13, sd = 0.29) relative to incongruent trials (mean = 1.18, sd = 0.31), and color-word location was [*F*_(1.7, 241.4)_ = 184.4, MSE < 0.01, $\eta_{p}^{2}$ = 0.6, *p* < 0.001]. Tukey-corrected pairwise comparisons of between different color-word locations revealed slower reaction times in the target location (mean = 1.22, sd = 0.34) than the background (mean = 1.12, sd = 0.28; mean difference = 0.10, SE = 0.01, *p* < 0.001) and distractor locations (mean = 1.13, sd = 0.28; mean difference = 0.09, SE = 0.01, *p* < 0.001). The difference between background and distractor was not significant (mean difference = 0.00, SE = 0.00, *p* = 0.82).

These main effects, however, were qualified by a significant interaction between age and location [*F*_(1.7, 241.4)_ = 33.6, MSE < 0.01, $\eta_{p}^{2}$ = 0.2, *p* < 0.001]. *Post-hoc* analysis indicated that younger adults were faster than older adults in all location conditions (*ps <* 0.001). Separate comparisons of reaction times between different location conditions for older and younger adults indicated that in both age-groups, reaction times were slower in the target location than in the background and distractor locations (*ps <* 0.001). Reaction times were not significantly different between background and distractor location conditions in either of the two age-groups (*ps >* 0.1).

Further, the interaction between location and congruency was also significant [*F*_(1.7, 243.7)_ = 88.0, MSE < 0.01, $\eta_{p}^{2}$ = 0.4, *p* < 0.001]. Comparing congruent and incongruent trials across different location conditions using Tukey-corrected comparisons indicated that reaction times were slower in incongruent than congruent trials only in the target condition (*p* < 0.001). There was no difference between congruent and incongruent trials in either the distractor or background location conditions (*ps >* 0.1). On the other hand, separate comparisons of reaction time between location conditions in congruent and incongruent conditions revealed in both types of trials, reaction times were slower in the target than in the background and distractor locations (*ps <* 0.001). Reaction times were not significantly different between background and distractor location conditions in either of the congruency conditions (*ps >* 0.5).

The two-way interaction between age and location, as well as the two-way interaction between location and congruency were qualified by a significant three-way interaction between location, congruency, and age [*F*_(1.7, 243.7)_ = 5.6, MSE < 0.01, $\eta_{p}^{2}$ = 0.4, *p* < 0.01]. Tukey-corrected comparisons revealed significantly slower reaction times in older adults in all location and congruency conditions (*ps <* 0.001). Incongruent trials induced slower reaction times in both younger and older adults only when the color-words were presented in the target shape (*ps <* 0.001) and not when the color-words were presented in the distractor or the background (*ps >* 0.05). In younger adults, target location induced slower reaction times than did distractor and background locations, but only for incongruent trials (*ps <* 0.001). In older adults, on the other hand, target location induced slower reaction time than distractor and background locations for both congruent and incongruent conditions (*ps <* 0.001). All other comparisons were not significant (for complete results, see supplementary materials).

Greenhouse–Geisser corrected ANOVA analysis with reaction time as the dependent variable, age-group and sex as between-subject factors and congruency and color-word location as the within-subject factors, revealed no significant effect or interaction of sex (see supplementary materials).

### Dual-stage two-phase (DSTP) model of the shape Stroop effect

#### Height of response selection boundary

**Figure 4:**
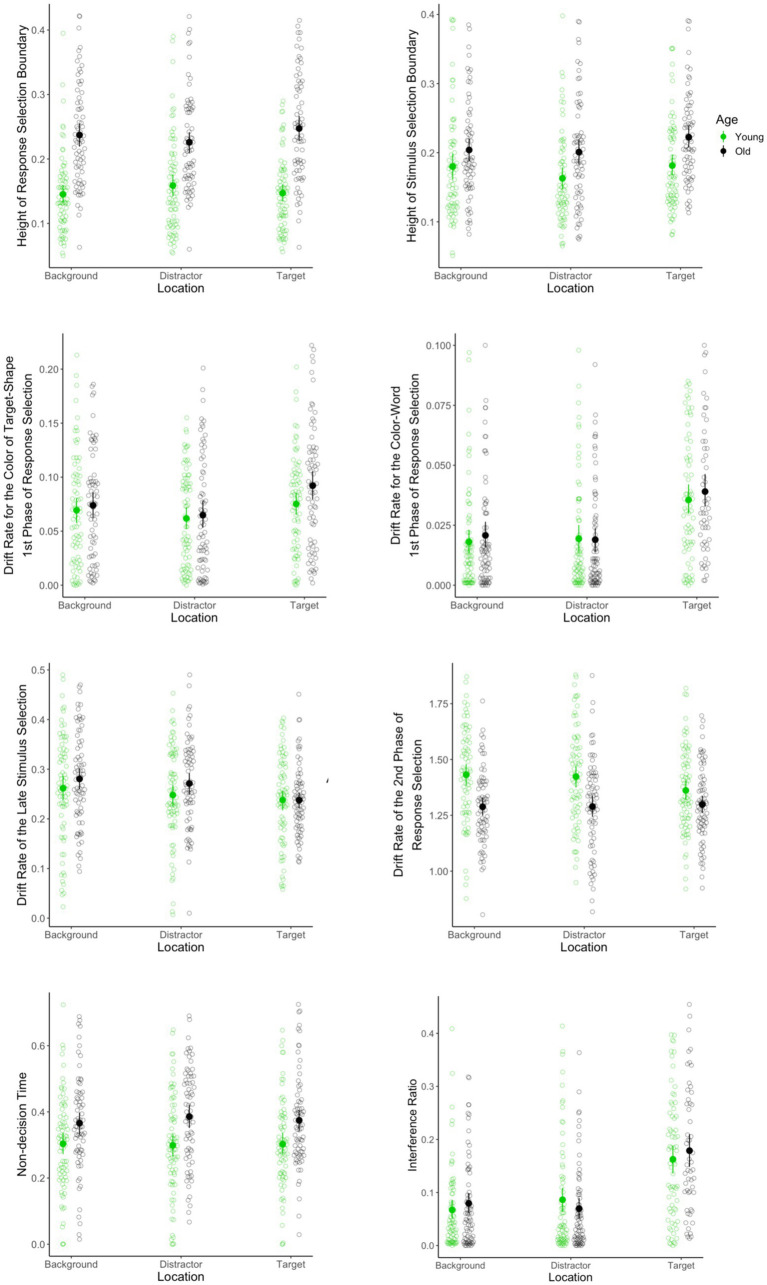
Parameters of the DSTP model fit for younger and older adults as a function of word location. Congruency is incorporated into the model by including congruent vs. incongruent trials as a factor in the fitted DSTP models.

Older adults adopted a higher response selection boundary than did younger adults [$F_{\mathrm{Age}}$(137, 1) = 77.4, *p* < 0.001], see Figure 4. The interaction between age and color-word location was significant [$F_{\mathrm{Age}*\text{Location}}$(268, 2) = 3.4, *p* = 0.03]. Further evaluation of the interaction effect revealed that older adults had higher response selection boundaries in all of the color-word locations (*p*s < 0.001). Contrasting the height of response selection boundaries between different color-word locations within each group revealed a significant difference between target and distractor location in older adults (*p* = 0.04): older adults adopted a higher response selection boundary in the target location (M = 0.256, sd = 0.093) relative to the distractor location (M = 0.235, sd = 0.94). Other simple effects were not significant (*p*s > 0.1).

#### Height of stimulus selection boundary

As is shown in Figure 4, the main effect of age was significant, such that older adults adopted a higher stimulus selection boundary [$F_{\mathrm{Age}}$(129, 1) = 16.9, *p <* 0.001]. The main effect of location was also significant [$F_{\text{Location}}$(258, 2) = 5.4, *p* = 0.005]. *Post-hoc* pairwise comparisons using Tukey correction, however, revealed significant difference between target and distractor location (*p* < 0.001) and no significant difference between target and background and no significant difference between background and distraction color-word locations (*p*s ≥ 0.1). Finally, the interaction between age and color-word location was not significant [$F_{\mathrm{Age}*\text{Location}}$(254.5, 2) = 0.4, *p* = 0.68].

#### Drift rate the color of the target-shape during the 1st phase of response selection

The main effect of age was not significant [$F_{\mathrm{Age}}$(141, 1) = 1.3, *p* = 0.25]. The main effect of location, however, was significant [$F_{\text{Location}}$(279, 2) = 6.8, *p* = 0.001]. Planned comparisons using Tukey correction revealed that the estimated $\mu_{\text{relevant}}$ was higher in the target than the distractor location (*p* < 0.001). Other *post-hoc* pairwise comparisons did not yield significant results (*p*s > 0.05). Finally, the interaction between age and the color-word location was not significant [$F_{\mathrm{Age}*\text{Location}}$(279, 2) = 0.6, *p* = 0.536]. Results are shown in Figure 4.

#### Drift rate for the color-word during the 1st phase of response selection

The main effect of age was not significant [$F_{\mathrm{Age}}$(119, 1) = 0.1, *p* = 0.71]. The main effect of location, however, was significant [$F_{\text{Location}}$(226.7, 1.9) = 33.5, *p* < 0.001]. Planned comparisons using Tukey correction revealed that the estimated $\mu_{\text{irrelevant}}$ was higher when color-words were in the target relative to both the distractor (*p <* 0.001) and background locations (*p* < 0.001). The difference between distractor and background location was not significant (*p* = 0.96). Finally, the interaction between age and color-word location was not significant [$F_{\mathrm{Age}*\text{Location}}$(226.7, 1.9) = 0.7, *p* = 0.5]. Results are shown in Figure 4.

#### Drift rate of the of the late stimulus selection

The main effect of age was not significant [$F_{\mathrm{Age}}$(142, 1) = 0.9, *p* = 0.355]. The main effect of location, however, was significant [$F_{\text{Location}}$(282.7, 2) = 16.1, *p* < 0.001], as is shown in Figure 4. Planned comparisons using Tukey correction revealed that the estimated $\mu_{\mathrm{ss}}$ was higher in the target than the background (*p <* 0.001) and distractor locations (*p <* 0.001). *Post-hoc* pairwise comparison between background and distractor did not yield significant results (*p*s > 0.1). Finally, the interaction between age and the color-word location was not significant [$F_{\mathrm{Age}*\text{Location}}$(282.7, 2) = 2.6, *p* = 0.078].

#### Drift rate of the of the second phase of response selection

As is shown in Figure 4, the main effect of age was significant, such that older adults had a slower rate of evidence accumulation in the second phase of response selection [$F_{\mathrm{Age}}$(140, 1) = 27.1, *p* < 0.001]. In contrast, neither the main effect of color-word location [$F_{\text{Location}}$(275.3, 2) = 1.3, *p* = 0.282] nor the interaction between age and location reached the significant level [$F_{\mathrm{Age}*\text{Location}}$(275.3, 2) = 1.9, *p* = 0.156].

#### Interference ratio index

The main effect of age was not significant [$F_{\mathrm{Age}}$(117, 1) = 0.00, *p* = 0.864]. The main effect of location, however, was significant [$F_{\text{Location}}$(206, 1.8) = 45.5, *p* < 0.001], as is shown in Figure 4. Planned comparisons using Tukey correction revealed that the estimated $\mu_{\text{irrelevant}}/\mu_{\mathrm{ss}}$ was higher in the target than both distractor (*p* < 0.001) and background locations (*p* < 0.001). The difference between distractor and background location was not significant (*p* = 0.72). Finally, the interaction between age and location was not significant [$F_{\mathrm{Age}*\text{Location}}$(206, 1.8) = 1.5, *p* = 0.22].

#### Non-decision time

The main effect of age was significant, such that older adults had longer non-decision times than did younger adults [i.e., encoding; $F_{\mathrm{Age}}$(140, 1) = 10.6, *p* = 0.001], see Figure 4. In contrast, neither the main effect of location [$F_{\text{Location}}$(269.3, 1.9) = 0.4, *p* = 0.66] nor the interaction between age and color-word location reached significance [$F_{\mathrm{Age}*\text{Location}}$(269.3, 1.9) = 1.00, *p* = 0.35].

### Overall summary of key findings

In summary, we found that, in comparison to younger adults, older adults (1) were more accurate, (2) had an equivalent level of Stroop effect when color-words were presented in the target shape, as revealed by similar reaction time pattern between younger and older adults (3) had intact response inhibition processes involved in different stages of selective attention, as revealed by comparable rates of evidence accumulation toward the color-word and the shape color in the first phase of stimulus selection processes, as well as comparable rates of evidence accumulation of late stimulus selection processes, (4) had more conservative decisional and attentional biases and therefore were more cautious, and (5) had a lower rate of accumulation of evidence in the second phase of response selection.

## General discussion

The purpose of this study was to evaluate the influence of age on attentional facilitation and response inhibition, and to understand how processes involved in different stages of selective attention may contribute to these potential age-related differences. To this end, we asked younger and older participants to perform a variant of the Stroop task called the Shape Stroop task, in which they had to name the color of a shape occluded by or occluding a shape of a different color while also ignoring congruent or incongruent Stroop color-words presented in either the target shape, the distractor shape or in the background.

We found that older adults were more likely to select a correct response in both congruent and incongruent trials. That is, they had higher accuracy rates than did younger adults. On the other hand, older adults were slower than younger adults in all location and congruency conditions. These findings replicate those finding age-related differences in speed-accuracy tradeoffs, whereby older adults have been shown to prioritize accuracy at the cost of speed (Salthouse, 1979). Smith and Brewer (1995), for example, showed that older adults adopt more conservative response strategies that result in slower RTs, but benefit accuracy. However, despite slower reaction times overall, we found age-invariance when comparing response times for congruent and incongruent trials. In both age-groups, incongruent trials induced slower reaction times than did congruent trials, but only when the color-words were presented in the target condition. Collectively, these results suggest that both younger and older adults are prone to Stroop effects when distracting (incongruent) information is presented in a foveal (target) location, and that the magnitude of this Stroop effect is age-invariant. Although this pattern of results is compatible with the results of previous studies of Shape Stroop in younger adults (Wühr, 2007; Wühr and Frings, 2008, 2009; Wühr and Waszak, 2003) and with Stroop effects that account for age-related processing speed differences (Verhaeghen and De Meersman, 1998; also see Salthouse, 1996), they are in contrast to some studies showing age-related differences in the Stroop effect (Bugg et al., 2007; Houx et al., 1993; Ludwig et al., 2010; Troyer et al., 2006; Van der Elst et al., 2006). For example, Bugg et al. (2007) showed that after accounting for age-related slowing, older adults experience a higher level of Stroop interference.

We also predicted that older adults would show slower responses/larger Stroop interference effects for incongruent trials in the distractor and background conditions relative to younger adults. This effect has been demonstrated by Geerligs et al. (2014) who reported that older adults may be more prone to interference from non-foveal information and have difficulties inhibiting irrelevant information when there is a spatial separation between the target and the irrelevant information. In contrast to these results, we found no evidence of older adults’ inefficiency in the inhibition of irrelevant information when there is a spatial separation between the target and the irrelevant information.

Although younger and older adults showed a similar pattern of results in the Shape Stroop task, it is possible that there may still exist age-related differences in the decision-making processes that led to these group-level results. That is, performance may depend on variations in early and/or late attention processes or changes in response strategies across different conditions. According to diffusion models, older adults often set wider decision boundaries than do younger adults. That is, they need to accumulate more evidence before making a decision, a finding which is consistent with an accuracy-over-speed strategy (Heitz, 2014; Ratcliff et al., 2007; Starns and Ratcliff, 2010).

To investigate and tease apart the influence of such factors and processes on performance, we modeled the data using the DSTP model (Hübner et al., 2010; Hübner and Pelzer, 2020). We found that older adults had higher response and stimulus selection boundaries than did younger adults. These results suggest that when there is a conflict, older adults are more cautious and require more evidence to make a decision. This is in line with the results of earlier studies showing that in the presence of interfering information or in a difficult task, older adults tend to spend additional time evaluating the contrasting information in order to make a correct response (Ratcliff and McKoon, 2015; Servant and Evans, 2020; Spieler et al., 1996). In addition, older adults showed a slower rate of evidence accumulation in the second phase of response selection relative to younger adults. This finding indicates that older adults’ response selection processing, after resolving a conflict, is affected in the Shape Stroop task. Based on this, older adults may have difficulties in response selection processing that is initiated after conflicts have been resolved. Moreover, we found that older adults had longer non-decision times, indicating older adults’ difficulties at encoding of the stimuli and initiating a behavioral response. Together, these results point to specific deficits in selective attention in aging, such that older adults are more conservative than younger adults and require more evidence to make a decision and to attend to a stimulus for further processing. This pattern of results supports the notion that older adults tend to prioritize accuracy over faster reaction time. This is in line with studies showing older adults’ tendency to adopt more conservative response strategies and prioritizing accuracy at the cost of speed (e.g., Smith and Brewer, 1995; also see Salthouse, 1979).

On the other hand, older and younger adults exhibited comparable rates of evidence accumulation toward the color-word and the shape color in the first phase of stimulus selection processes, as well as comparable rates of evidence accumulation of late stimulus selection processes. The finding of age-invariance in the rate of evidence accumulation of late stimulus selection, as well as age-invariance in the rate of evidence accumulation for the color-word in the first phase of response selection, suggests that inhibitory processes involved in selective attention may not be affected by aging, a possibility that is further supported by the finding of age-invariance in the interference ratio index, which indexes the magnitude of interference that is induced by the color-word. These results are in line with the results of DDM studies showing age-invariance in rate of evidence accumulation in memory recognition, perceptual discrimination, and lexical decision tasks (e.g., McKoon and Ratcliff, 2012; Ratcliff et al., 2006; Ratcliff et al., 2004b; Ratcliff et al., 2004a).

In addition to theoretical implications for perceptual inhibition in older adults, the current study also sheds light on the computational bases of the Stroop effect within the paradigm of the Shape Stroop task (Wühr, 2007; Wühr and Frings, 2008, 2009; Wühr and Waszak, 2003). More specifically, our results indicate that presenting color-words in the target shape induces (1) a higher rate of evidence accumulation for the shape-color than the distractor condition in the first phase of response selection, (2) a higher drift rate of evidence accumulation for the color-word than the distractor and background conditions in the first phase of response selection, (3) a lower rate of evidence accumulation of the late stimulus selection than the background condition and induces (4) stronger interference than the distractor and background conditions as indexed by interference ratio index. Hence, our results suggest that presenting color-words in the target enhances the amplification of the shape-color, but also diminishes inhibitory processes involved in early and late stages of selective attention. Therefore, stronger interference results from presenting color-words in the target than in the distractor or in the background. It should be noted that these effects were not dependent on the age of participants or the response strategies they adopted.

### Limitations

While this study provides a detailed examination of age-related differences in attentional and inhibitory processes and the potential causes of these differences, there are several limitations. First, participants completed the task without supervision in their homes. Thus, it is possible that they encountered distractions that they may not have had they been tested in a laboratory setting. However, completion rates for the tasks were not different between age-groups, partially mitigating this concern. Second, it is possible that computer literacy or age-related differences in familiarity with technology could have affected our results. While we cannot rule this out, the participants had completed numerous other computerized tests as part of other studies and had to have access to their own computer to download the software to run the SOFIA tests at home. Further, we found age-invariant (or indeed older adults outperforming younger adults) measures of accuracy in the task, as well as not age-related differences in the control condition, in which we trained participants on the stimulus–response mapping.

## Data Availability

The datasets presented in this study can be found in online repositories. The names of the repository/repositories and accession number(s) can be found in the article/supplementary material.
